# Coadsorption Interfered CO Oxidation over Atomically Dispersed Au on *h*-BN

**DOI:** 10.3390/molecules27113627

**Published:** 2022-06-05

**Authors:** Xin Liu, Xin Zhang, Changgong Meng

**Affiliations:** State Key Laboratory of Fine Chemicals, Department of Chemistry, Dalian University of Technology, Dalian 116024, China; xin0123@mail.dlut.edu.cn

**Keywords:** single atom catalysis, Au, CO oxidation, reaction conditions, *h*-BN, first principles

## Abstract

Similar to the metal centers in biocatalysis and homogeneous catalysis, the metal species in single atom catalysts (SACs) are charged, atomically dispersed and stabilized by support and substrate. The reaction condition dependent catalytic performance of SACs has long been realized, but seldom investigated before. We investigated CO oxidation pathways over SACs in reaction conditions using atomically dispersed Au on *h*-BN (AuBN) as a model with extensive first-principles-based calculations. We demonstrated that the adsorption of reactants, namely CO, O_2_ and CO_2_, and their coadsorption with reaction species on AuBN would be condition dependent, leading to various reaction species with different reactivity and impact the CO conversion. Specifically, the revised Langmuir–Hinshelwood pathway with the CO-mediated activation of O_2_ and dissociation of cyclic peroxide intermediate followed by the Eley–Rideal type reduction is dominant at high temperatures, while the coadsorbed CO-mediated dissociation of peroxide intermediate becomes plausible at low temperatures and high CO partial pressures. Carbonate species would also form in existence of CO_2_, react with coadsorbed CO and benefit the conversion. The findings highlight the origin of the condition-dependent CO oxidation performance of SACs in detailed conditions and may help to rationalize the current understanding of the superior catalytic performance of SACs.

## 1. Introduction

Single atom catalysis is emerging and growing as a new frontier in catalysis [1,2]. Similar to the metal centers in enzymes and transition metal complexes in biocatalysis and homogeneous catalysis, the transition metal species in SACs are charged and atomically dispersed on the support [3,4]. In reaction conditions, the thermodynamics-driven adsorption and reaction of substrates would change the oxidation state [5] and coordination of metal species drastically [6], and may stabilize metal species in an atomically dispersed form or promote their aggregation or redispersion [7]. This feature of transition metal species in reaction conditions were reported for Au [8,9,10,11,12], Pd [13], and Rh [14] -based SACs, etc. Apart from the potential aggregration/redispersion of metal species, the adsorption/coadsorption of the substrate may also impact the thermodynamics and kinetics for the evolution of reaction species and may lead to a switch of the pathways for substrate conversion with reactions conditions, such as temperature, substrate concentrations (partial pressures), [15,16,17,18,19] etc. For these complexities, the impact of the reaction condition to active sites and active species was seldom investigated [19].

Low temperature catalytic oxidations of CO oxidation to CO_2_ are the key components in many industrial chemical processes, such as the water–gas–shift reaction [20], CO preferential oxidation in H_2_ rich stream (CO-PROX) [21], exhaust gas control [22], etc. Apart from the practical applications, CO oxidation is widely used as a prototypical reaction for fundamental investigations [23] and it is also the first catalytic reaction realized on Pt_1_/FeO_x_ [24]. Continuous efforts have been made to develop novel CO oxidation catalysts that are efficient at low temperatures [22]. Among transition metals used for CO oxidation, Au is special, as it is chemically inert in the bulk phase, and is highly active when is downsized to a sub-nano scale [25,26]. Since the supported Au nanoparticles were found active for CO oxidation in the 1980s, tremendous efforts have been put to understand the active species and mechanism pathways for the Au-catalyzed CO oxidation in operating conditions [26]. For many of these cases, the charged Au atoms, formed dynamically in reaction conditions, were proposed to be the active sites [5,10,11,12,19,27,28]. Furthermore, it has been demonstrated that Au SACs are highly active for many reactions [29], such as the water–gas–shift [27], methanol steam reforming [30], epoxidation of ethylene, [31] acetylene hydrochlorination [32], selective hydrogenation [33], ethanol dehydrogenation reactions [34], CO oxidation [11,19,35,36,37,38,39], etc.

Hexagonal boron nitride (*h*-BN) is a 2D material with graphene-like planar structure [40]. Defects, such as vacancies, etc., that are capable of modulating electronic properties of *h*-BN, can be created by electron beam irradiation, solvent and gas exfoliation, ball milling [41,42], etc. The metal/defective *h*-BN interaction stabilizes the metal species and tailors their reactivity [43,44,45,46,47,48,49]. Several *h*-BN-supported SACs have been proposed effective for reactions of practical interest, such as the electroreduction of N_2_ and CO_2_ [47,48,49,50], hydrogenation of Cinnamaldehyde [51], dehydrogenation of light alkanes [52] and CO oxidation [53,54] etc. However, the active site and active species in reaction conditions for these processes have not been addressed.

Recently, we theoretically explored the reaction network for CO oxidation over Pd_1_ and Fe_1_ SACs on graphene and highlighted the vital role of the thermostability of reaction species in determining CO conversion and reaction pathways [55,56]. In this work, we focus on CO oxidation over AuBN to highlight the impact of coadsorbed substrates to the formation and evolution of surface species on SACs in reaction conditions with extensive first-principles-based calculations. We expect the finding would be helpful for the discovery of new reaction pathways and a rationalized understanding to the observed superior performance of SACs.

## 2. Theoretical Methods

All the first-principles-based calculations were performed using the GGA-PBE functional [57] with the DSPP pseudopotential [58] and DNP basis set [59], as implemented in DMol^3^ [60,61]. CO oxidation over AuBN was investigated in a 6 × 6 supercell of *h*-BN. The Brillouin Zone was sampled with a Γ-centered 4 × 4 × 1 *k*-point grid [62]. The global orbital cutoff was set as 4.50 Å and the convergence criterion for energy and forces were 3 × 10^−4^ eV and 5 × 10^−2^ eV/Å, respectively. The transition states (TSs) were determined with synchronous transition methods, and further optimized and confirmed with frequency analysis, so that the only negative frequency is on the bond formation/dissociation direction [63]. The Hirshfeld scheme was adapted for population analysis [64]. With the above setup, the bulk lattice parameter of face-center-cubic Au and *h*-BN were calculated as 4.20 and 2.52 Ǻ, respectively [65,66].

The formation free energy of a reaction species with stoichiometry of $Au{\left(CO\right)}_{x}{\left(O_{2}\right)}_{y}$ over AuBN, under CO oxidation conditions, was calculated as: $\Delta G=G_{Au{\left(CO\right)}_{x}{\left(O_{2}\right)}_{y}}-E_{Au}-G_{BN}-x\times G_{CO}-y\times G_{O_{2}}+z\times G_{CO_{2}}$. Here, x, y and z are the stoichiometry for CO, O_2_ and CO_2_ involved in the formation of $Au{\left(CO\right)}_{x}{\left(O_{2}\right)}_{y}$, respectively, $G_{Au{\left(CO\right)}_{x}{\left(O_{2}\right)}_{y}}$ and $G_{BN}\;$ are the free energy of $Au{\left(CO\right)}_{x}{\left(O_{2}\right)}_{y}$ and B-vacancy *h*-BN, respectively, $E_{Au}$ is the energy of an Au atom, $G_{CO}$ and $G_{O_{2}}$ are free energy of gas molecules calculated as $G_{gas}\left(T,p\right)=E_{gas}^{e}+\Delta \mu_{gas}\left(T,p^{0}\right)+k_{B}Tln\left(\frac{p}{p^{0}}\right)$, where $E_{gas}^{e}$ is the calculated total energy of the gas molecule and $\Delta \mu_{gas}\left(T,p^{0}\right)$ is derived from calculated partition functions of the gas molecule. The reaction conditions described by T,p were previously used to characterize the performance of SACs in CO oxidation [67].

## 3. Results and Discussions

### 3.1. Thermodynamics Analysis of Potential Reaction Species

**Figure 1:**
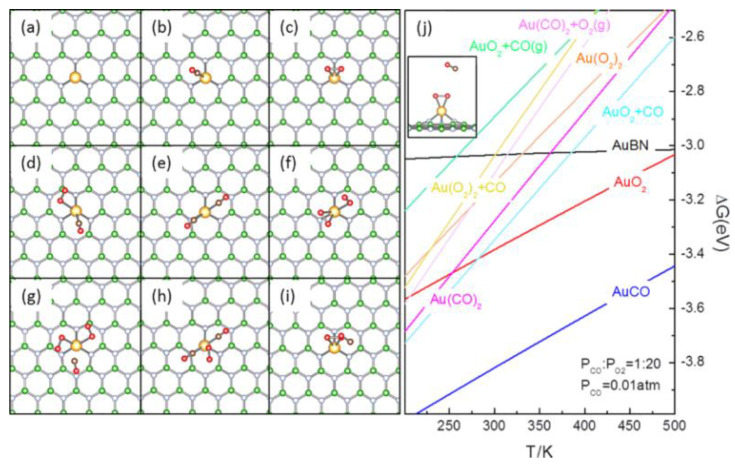
The calculated atomic structures (**a**–**i**) and temperature dependence of ∆G (j) for the potential reaction species formed over AuBN in CO oxidation. Please see the context for the notation of structures. ∆G was calculated with respect to B-vacancy on *h*-BN, Au atom at P_CO_ = 0.01 atm and P_CO_/P_O_2__ = 1:20. The inset in (**j**) is the sideview of (**i**). In (**a**–**i**), the B, N, C, O and Au atoms are in green, light blue, brown, red and gold, respectively.

We firstly evaluated the formation of Gibbs free energies (ΔG) of all the potential reactants at various temperatures (T) at P_CO_:P_O2_ = 1:20 and P_CO_ = 0.01 atm (Figure 1), which are commonly used to characterize the CO oxidation performance of a catalyst [67].

The calculated binding energy (E_b_) of the Au atom on the B-vacancy of *h*-BN (AuBN, Figure 1a) in a triplet ground state is −3.54 eV and is comparable to calculated (−2.90 eV), previous theoretical (−3.03 eV) [68] and experimental results of bulk Au (−3.81 eV) [69], suggesting AuBN would be plausible over other potential Au deposition structures [52,53]. Considering the poor E_b_ of the Au atom (−0.11 and −0.10 eV, respectively, on top of N and B atoms on pristine *h*-BN), the outward diffusion of Au from AuBN would be highly endothermic (>3 eV), so it is hard to expect that the Au atom would diffuse away. In AuBN, the Au-N distances are ~2.08 Å and Au is 0.45|e| positively charged. These are in excellent agreement with the reported X-ray photoelectron spectroscopy of Au SACs [24,34,35,70], transition metal SACs on *h*-BN and Au/*h*-BN nanocomposites, where the Au and TM atoms are confirmed to be positively charged as reaction centers [41,51,71]. The thermodynamics data (Figure 1j) demonstrated that CO adsorbed on AuBN (AuCO, Figure 1b, E_ad_ = −1.38 eV) is the most plausible at temperatures from 200 to 500 K. In AuCO, Au is 0.36 |e| positively charged, and C-O bond is in the direction nearly reverse to one of the Au-N bonds at Au-C distance of 1.93 Å and is slightly stretched (1.16 Å). As for the adsorption of O_2_ (AuO_2_, Figure 1c, E_ad_ =−0.90 eV), the O-O bond is elongated to 1.33 Å, parallel to the *h*-BN surface, and is nearly vertical to one of the N-Au bonds, forming an undercoordinated octahedral. Au is 0.53 |e| positively charged and the spin density is localized on Au and O atoms. ∆G of other potential species (Figure 1d–i) were also collected (Figure 1j). ∆G of AuO_2_ and the coadsorption of CO and O_2_ (AuO_2_ + CO, Figure 1d, E_ad_ = −1.49 eV) stand right above that of AuCO, are negative from 250 to 500 K and intersect each other at ~280 K, demonstrating that AuO_2_ + CO may form from AuCO via coadsorption with O_2_ or by an exchange of adsorbates. ∆G of other coadsorption species, such as coadsorption of 2 CO (Au(CO)_2_, Figure 1e), 2 O_2_ (Au(O_2_)_2_, Figure 1f) and the van de Waals complexes formed between adsorbed O_2_/CO and gaseous molecules, such as Au(O_2_)_2_ + CO(g), Au(CO)_2_ + O_2_(g) and AuO_2_ + CO(g) (Figure 1g–i), etc., are much higher in ∆G than those for AuO_2_, AuCO and AuO_2_ + CO (Figure 1j). Though these species may potentially exist, their coverage would be much lower than those for the AuCO, AuO_2_ and AuO_2_ + CO, and they would evolve into these more stable species by adsorbate desorption or exchange. Our results (Figure 1) clearly indicate that AuCO (Figure 1b) is the most plausible reaction species in the 200–500 K temperature range, under CO oxidation conditions. We also investigated the impact of CO and O_2_ chemical potential on the relative stability in terms of the ∆G of these reaction species by varying the CO and O_2_ partial pressure in the range of 0.01–0.2 atm in the same temperature interval. Though the calculated values of ∆G of these identified major reactants, namely AuCO, AuO_2_, AuO_2_ + CO and Au(CO)_2_ (Figure 1b–e) may shift due to the variation of reaction conditions, AuCO is still the most plausible, considering that the ∆G of AuO_2_ and AuO_2_ + CO remain right above that of AuCO. This is in reasonable agreement with the experimental finding that the active reaction species in SAC-catalyzed CO oxidation all originate from positively charged or even oxidized metal atoms [72,73,74,75]. We also investigated the potential impact of van der Waals interactions on the relative stability of these species within the same temperature and partial pressure range, and yielded exactly the same finding. The calculated stretching frequency of AuCO (Figure 1b) is slightly redshifted from that of gaseous CO, i.e., from 2143 cm^−1^ to 2040 cm^−1^, and further shifted to 2115 cm^−1^ in AuO_2_ + CO (Figure 1d). The calculated CO stretching frequencies correlate well with the calculated charge on Au in AuBN, AuCO and AuO_2_ + CO, confirming the positively charged nature of the Au atom in AuBN. Similar redshifts of the C = O stretching frequency have been reported for CO oxidation over Au and Pd-based SACs and nanoparticles catalysts [20,74,75,76,77]. Based on the consistent evidence for the positively charged nature of AuBN (Figure 1a, AuBN), we moved further to investigate its performance in CO oxidation.

### 3.2. Revised LH Pathway for CO Oxidation over AuBN

**Figure 2:**
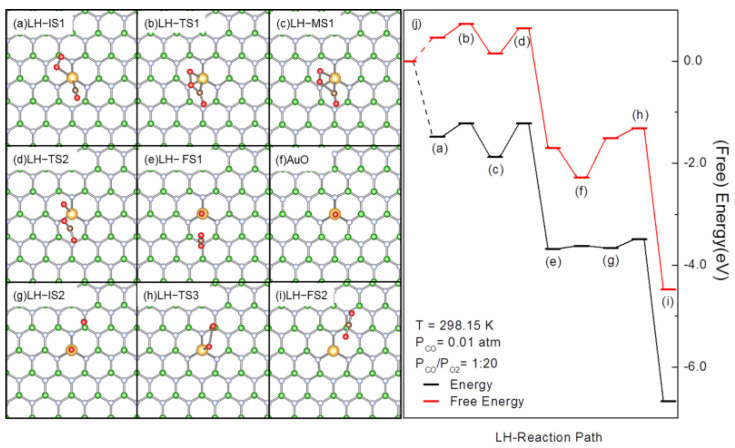
Optimized structures of the reaction species involved, including reaction intermediates, transition states and products (**a**–**i**), and (free) energy profiles (**j**) for CO oxidation over AuBN through the rLH pathway. In (**a**–**i**), the B, N, C and Au atoms are in green, light blue, brown and gold, respectively. In (**j**), the bracketed letters correspond to the structures shown in (**a**–**i**).

Based on experimental and theoretical results, several pathways have been proposed for the CO oxidation over SACs [22], which can be classified into two kinds according to the involvement of support during the reaction. Pristine *h*-BN is chemically inert to CO and O_2_. As shown in Figure 1a, the proposed AuBN has the B-vacancy on *h*-BN fully passivated, there is no other defect at the chemical bond distance from Au to stabilize CO, O_2_ or other reaction species. Therefore, the pathways that require support or defects on the support for stabilization and activation of CO or O_2_, and formation and stabilization of reaction species, such as the Mars-van-Krevelen type pathway and its variants that requires support oxygen to init [24,70], support-promoted CO oxidation that needs support to stabilize reactants [78], etc., are not applicable on AuBN. Another kind of CO oxidation pathway inherits the merit of Langmuir–Hinshelwood (LH) and Eley–Rideal(ER) type pathways derived from bulk metals [79,80], involving the adsorption and activation of one reactant (ER), either CO or O_2_, or both (LH) only on the metal species. We recently investigated CO oxidation over the defect-stabilized Fe and Pd SACs on graphene and demonstrated that the coadsorption of CO and O_2_ would be more plausible on these SACs over van de Waals complexes and the coadsorption of 2 CO to initiate CO oxidation on ER type pathways. Thus, it would be a major reaction species with a population superior to those van de Waals complexes and is the active species to initiate CO oxidation [55,56,81]. The calculated thermodynamics stability of reaction species on AuBN (Figure 1j) also demonstrated such a trend that AuO_2_ + CO is more plausible than AuO_2_ + CO(g) and Au(CO)_2_, though AuCO is the most plausible species in reaction conditions. This AuO_2_ + CO (Figure 1d) satisfies the requirements to initiate reaction on LH type pathways that both O_2_ and CO adsorb on AuBN and get activated for reactions. The first reported pathway of the LH type involves coadsorbed CO-assisted O_2_ activation through the formation and dissociation of peroxide (OCOO) species on the metal center (revised Langmuir–Hinshelwood pathway, rLH), and was originally proposed by Iglesia et al., and was then adapted as the pathway for the CO and ethylene oxidation over Au SAC [31,39,82]. We then started with the rLH pathway from AuO_2_ + CO (LH–IS1, Figure 2a).

The elementary steps on the rLH pathway for CO oxidation were investigated (Figure 2). The E_ad_ for AuO_2_ + CO (LH-IS1, Figure 2a, the same as Figure 1d) is −1.48 eV. In LH-IS1, Au-C(CO) and Au-O(O_2_) are both in directions reverse to Au-N bonds. Though the E_ad_ of CO and O_2_ are significant, they are repulsive to each other, making ΔG for the LH-IS1 formation (at 298.15 K, P_CO_ = 0.01 atm, P_CO_/P_O_2__ = 1:20) 0.46 eV higher than that of AuCO (Figure 1b). Considering the large exothermicity for the formation of LH-IS1 (~−3.00 eV), LH-IS1 may exist but with a smaller population. The variation of ∆G from AuCO to LH-IS1 also suggests that CO in AuO_2_ + CO (LH-IS1) would be active for subsequent reactions [76]. Driven by the electrostatic interaction between O(O_2_) (−0.06|e|) and C(CO) (+0.15 |e|), the O(O_2_) atom moves to interact with C(CO). By crossing a transition state (LH-TS1, Figure 2b) with energy and free energy barriers of 0.26 and 0.27 eV, respectively, a peroxide intermediate (LH-MS1, Figure 2c) is formed. The newly formed C-O bond stabilizes the reaction product and makes the formation of LH-MS1 slightly exothermic (∆G = −0.31 eV) with respect to LH-IS1. In LH-MS1, the O-O distance is elongated to 1.51 Å and is typical for O-O bonds in peroxides [83]. The C=O stretching frequency in LH-MS1 was calculated as 1748 cm^−1^, which falls in the higher range of experimentally reported values [73,74,75]. Due to the instability of the peroxide O-O bond, charge reorganization may take place within LH-MS1 for passing a TS(LH-TS2, Figure 2e) to form a CO_2_ molecule (LH-FS1, Figure 2f). This tendency for breaking the O-O bond and reorganizing the structure to form CO_2_ is well evidenced by the change of C = O distance from 1.72 to 1.18 Å, and the elongation of O-O and Au-C distances from 1.51 and 2.11 Å, respectively, to 3.35 and 4.27 Å, respectively. Charge transfer to C and 2 O(O_2_) atoms also takes place simultaneously. The calculated Hirshfeld charges on the C and 2 O(O_2_) increase from 0.09, −0.18, −0.05 |e|, respectively, in LH-MS1 to 0.10, −0.10 and −0.30 |e|, respectively, in LH-TS2. This charge transfer is further enhanced in LH-FS1, where the charges on C, O(CO_2_), O(Au) and Au are 0.28, −0.15, −0.30 and 0.56 |e|, respectively, implying the oxidation of Au in this process and the weak binding of CO_2_ to the Au = O center. This is in agreement with the calculated E_ad_ of CO_2_ in LH-FS1 of only −0.07 eV. The further desorption of CO_2_ leads to the formation of AuO (Figure 2f), which is more thermodynamically stable than LH-FS1. AuO will then form a van de Waals complex with gaseous CO (LH-IS2, Figure 2g). The calculated ∆E and ∆G between LH-FS1 and LH-IS2 are only 0.02 and 0.19 eV, respectively. The electrostatic interaction between C(CO) (+0.06 |e|) and O(Au)(−0.30|e|) stabilizes LH-IS2 and initiates the subsequent reaction with charge transfer from the 5σof CO to the π* on O(Au) for the reduction of Au. CO moves towards O(Au) in this process to reach the transition state (LH-TS3, Figure 2h), where the C-O(Au) distance decreases to 1.90 Å and the calculated Hirshfeld charge on C and O(Au) changes to 0.06 and −0.26 |e|, respectively, indicating a charge transfer from CO to O for the formation of the C=O bond. By crossing the energy barrier of 0.17 eV (LH-TS3), another CO_2_ is formed (LH-FS2, Figure 2i). CO_2_ desorption from AuCO_2_ would be easy even at a low temperature, as the calculated E_ad_ of CO_2_ is only −0.07 eV and thus closes the catalytic cycle for CO oxidation along the rLH pathway.

### 3.3. Interference of the Coadsorption of CO or O_2_ to CO Oxidation

**Figure 3:**
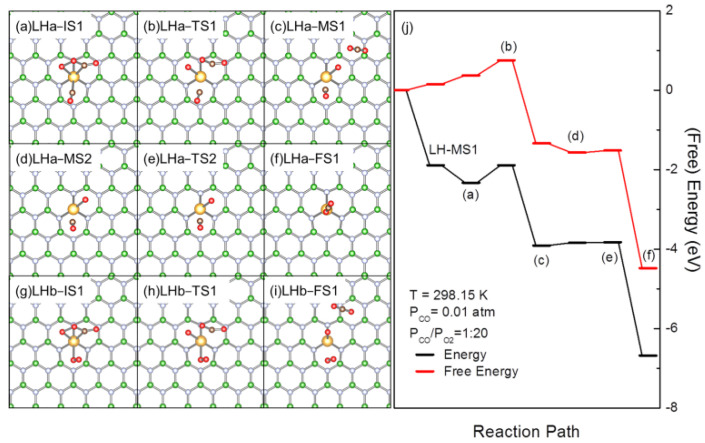
Optimized structures of the reaction species involved, including reaction intermediates, transition states and products (**a**–**i**), and (free) energy profiles (**j**) for CO oxidation over AuBN with the LHa and LHb pathways initiated with the coadsorption of CO or O_2_ with LH-MS1. In (**a**–**i**), the B, N, C and Au atoms are in green, light blue, brown and gold, respectively. In (**j**), the bracketed letters correspond to the structures shown in (**a**–**i**).

LH-MS1 is an important intermediate on the rLH pathway with a dissociation free energy barrier of 0.51 eV at 298.15 K when P_CO_ = 0.01 atm and P_CO_/P_O2_ = 1:20. Considering the high barrier for dissociation and a small barrier for formation, LH-MS1 would be one of the major reaction species [55]. A careful inspection of the structure shows that Au in LH-MS1 is not fully coordinated in a penta coordinated environment, implying potential coadsorption and further reactions with CO or O_2_ (Figure 3). The calculated E_ad_ for CO coadsorption with LH-MS1(LHa-IS1, Figure 3a) is −0.44 eV and ΔG is 0.22 eV (at 298.15 K, P_CO_ =0.01 atm, P_CO_/P_O2_ = 1:20). The variation of ∆G from LH-MS1 also suggests that the adsorbed CO would be reactive for subsequent reactions [55,76]. In LHa-IS1, Au is +0.61 |e| charged to interact with 3 N at a B-vacancy of *h*-BN, O and C of peroxide intermediate (OOCO) and the newly adsorbed CO. The calculated C = O stretching frequency in OOCO is blue shifted from 1748 cm^−1^ to 1811 cm^−1^, while that in CO is also blue shifted to 2125 cm^−1^. These correlate well with the charge on Au. OCOO dissociation and subsequent reactions was then investigated. As aforementioned, OCOO will dissociate to form CO_2_ and AuO with an energy barrier of 0.51 eV. The calculated energy barrier for OCOO dissociation in LHa-IS1 is 0.44 eV and is lowered by 0.07 eV. One may raise concern with the CO desorption during or prior to OCOO dissociation. The O(CO)-Au distance is decreased slightly from 2.09 (in LHa-IS1) to 2.05 Å in the corresponding TS (LHa-TS1, Figure 3b) and is further decreased to 2.02 Å in the product (LHa-MS1, Figure 3c) that is a coadsorption structure of atomic O and CO with a CO_2_ interacting electrostatically with O(Au). The continuous decrease in the Au-C distance during this process indicates that OCOO dissociation may not drive desorption of CO but stabilize it instead. After desorption of CO_2_ in LHa-MS1, further reactions may take place between coadsorbed CO and O in LHa-MS2 (Figure 3d). As mediated by Au, the coadsorbed O and CO combine together by crossing a TS (LHa-MS2, Figure 3e) with a reaction barrier of 0.01 eV and free energy barrier of 0.05 eV, to form another CO_2_ (LHa-FS1, Figure 3f). The calculated (free) energy barriers on the newly proposed pathway for the evolution of LHa-IS1 are much lower than those on the rLH pathway (Figure 2j and Figure 3j). The decreased reaction barrier provides direct evidence for the promotion effect of CO in OCOO dissociation.

Coadsorption of O_2_ with OCOO leads to the formation of LHb-IS1 (Figure 3g). The calculated E_ad_ of O_2_ is −0.15 eV and the corresponding ΔG is 0.41 eV. The weak adsorption of O_2_ can be attributed to the positive charged nature of the Au center in LH-MS1. The calculated O_2_ dissociation energy barrier and free energy barrier on AuBN is 1.79 and 1.78 eV, respectively, so AuBN is not capable of O_2_ dissociation. The reaction goes through a TS (LHb-TS1, Figure 3h) with a barrier of 1.00 eV and the free energy barrier of 0.92 eV, forming physisorbed O_2_ and CO_2_ around AuO (LHb-FS1, Figure 3i). As the OCOO dissociation barrier is increased to be much higher than E_ad_ (ΔG) for O_2_ adsorption, O_2_ desorption may take place with high priority and may not impact OCOO dissociation.

**Figure 4:**
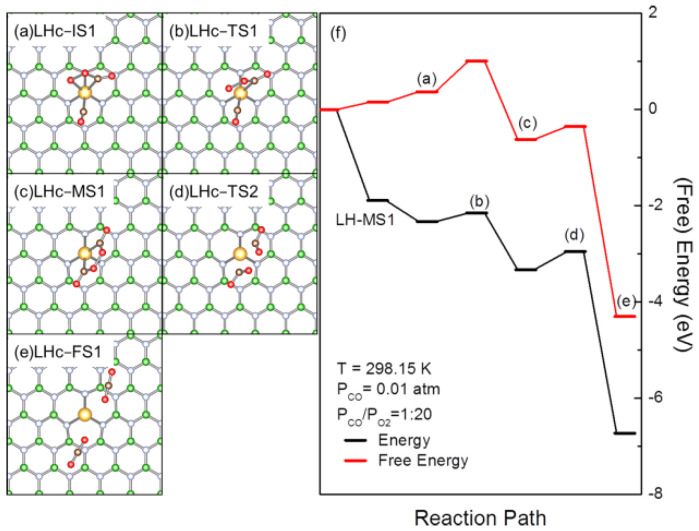
Optimized structures of the reaction species involved, including reaction intermediates, transition states and products (**a**–**e**), and (free) energy profiles (**f**) for CO oxidation over AuBN, with the LHc pathway initiated with the coadsorption of CO with LH-MS1. In (**a**–**e**), the B, N, C and Au atoms are in green, light blue, brown and gold, respectively. In (**f**), the bracketed letters correspond to the structures shown in (**a**–**e**).

Apart from promoting the dissociation of OCOO, the coadsorbed CO may also react with OCOO. In LHc-IS1 (Figure 4a), Au is highly positively charged (+0.61|e|), O bound to Au is negatively charged (−0.20 |e|) and C(CO) is also positively charged (+0.19 |e|). CO may interact electrostatically with O(Au) to reach a TS (LHc-TS1, Figure 4b) with energy and free energy barriers of 0.18 and 0.64 eV, respectively, for the formation of OCOOCO intermediate (LHb-MS1, Figure 4c) that was previously proposed on a three-molecule ER(ER3) type pathway for CO oxidation [53]. In LHb-TS1, OCOO is distorted to facilitate the O(Au)-C(CO) interaction, with the increase in the O(Au)-Au and decrease in O(Au)-C(CO) distance, showing the tendency for the formation of the Au-C bond at the expense of the Au-O bond. The corresponding energy and free energy barriers are 0.18 and 0.64 eV, respectively. As a result of these interactions, CO is inserted into the O-Au bond forming a planar OCOOCO intermediate nearly vertical to one of the interfacial Au-N bonds (LHc-MS1). Due to the instability of the peroxo O-O bond, LHc-MS1 may undergo dissociation by breaking the O-O and Au-C bonds in the corresponding TS (LHc-TS2, Figure 4d) with energy and free energy barriers of 0.38 and 0.27 eV, respectively, to form 2 CO_2_ physisorbed at AuBN (LHc-FS1, Figure 4e).

As Au in LHb-MS1 is also pentacoordinated, coadsorption of CO or O_2_ may also take place. The impact of coadsorbed CO and O_2_ for the further evolution of LHc-MS1 was also investigated. Both CO and O_2_ cannot react with OCOOCO intermediate, they will retain and adsorb on AuBN after the dissociation of LHc-MS1. As Au is already positively charged in LHc-MS1, the calculated E_ad for_ CO and O_2_ coadsorption are -0.21 and −0.09 eV, respectively, while ΔG are 0.52 and 0.46 eV, respectively. This is similar to the aforementioned coadsorption of CO or O_2_ with OOCO. With the coadsorption of reactants, the energy and free energy barriers for OCOOCO dissociation change to 0.49 and 0.28 eV, respectively, for CO and 0.19 and 0.15 eV, respectively, for O_2_. The variation of the reaction barrier with coadsorption of CO or O_2_ once again provides direct evidence for the interference of coadsorbed CO or O_2_ to the evolution of reaction species and CO conversion.

**Figure 5:**
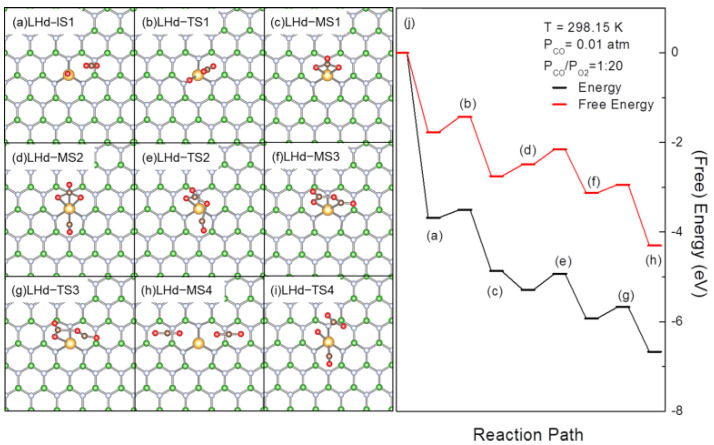
Optimized structures of the reaction species involved, including reaction intermediates, transition states and products (**a**–**i**), and (free) energy profiles (**j**) for CO oxidation over AuBN with the LHd pathway initiated with the coadsorption of CO with LH–MS1. In (**a**–**i**), the B, N, C and Au atoms are in green, light blue, brown and gold, respectively. In (**j**), the bracketed letters correspond to the structures shown in (**a**–**h**).

AuO is another important intermediate on the rLH pathway for CO oxidation. AuO is formed by the dissociation of LH-MS1 and may be consumed by direct reaction with gaseous CO [39]. In AuO, O is negatively charged (−0.30 |e|). Previously, such negatively charged O-containing species in mesoporous carbon materials were proposed as reactive CO_2_ binding sites [84]. The calculated CO_2_ E_ad_ for CO_2_ physisorption on AuO (LHd-IS1, Figure 5a) is −0.07 eV and the corresponding ΔG is 0.51 eV. These are in reasonable agreement with Pd, Fe and Au SACs on graphene [31,55,56]. In LHd-IS1, both the C-O bonds are nearly vertical to C(CO_2_)-O(Au) direction. Following the O(Au)-C(CO_2_) electrostatic interaction, CO_2_ moves to O(Au) by crossing a TS (LHd-TS1, Figure 5b) with a barrier of 0.18 eV and free energy barrier of 0.34 eV for the formation of carbonate (CO_3_, LHd-MS1, Figure 5c). The O(CO)-C-O angle also changes from 85 to ~94°, demonstrating that CO_2_ is activated to interact with both O(Au) and Au in this process. The interaction is further enhanced in LHd-MS1, as the O(C)-C-O(Au) is distorted to be ~120°. Au in AuBN is also oxidized during this process, as the charge on Au changes from 0.56 |e| to 0.64 |e| in LHd-MS1. The energy and free energy change for the formation of CO_3_ are −0.98 and −1.19 eV, respectively, while the calculated energy and free energy barriers for CO_3_ dissociation are 1.38 and 1.28 eV, respectively. Therefore, the direct dissociation of CO_3_ is rather demanding over AuBN. In this sense, CO_3_ formation is plausible by the reaction of gaseous CO_2_ with AuO. This pathway also connects the evolution of OCOO to thermodynamically more plausible CO_3_ species and solves the concern for formation and evolution of CO_3_ species in CO oxidation on SACs [73,74,75,85]. We then moved on to investigate the potential coadsorption with CO and whether CO may promote the conversion of CO_3_ [56]. The CO coadsorption leads to the formation of LHd-MS2 (Figure 5d). The calculated CO E_ad_ and ΔG are −0.42 and 0.16 eV, respectively. The small positive change of ΔG suggests coadsorbed CO would be highly reactive [55,76]. According to the charge distribution, the positively charged C(CO) may move to react with negatively charged O(Au) at the interface by crossing the TS (LHd-TS2, Figure 5e) with the energy barrier and free energy barriers of 0.36 and 0.34 eV, respectively, to reach the intermediate (LHd-MS3, Figure 5f), where CO is inserted into an Au-O bond forming a planar C_2_O_4_ species with an ultra-long C-O bond of 1.45 Å, which introduces instability into this structure. LHd-MS3 may further evolve by breaking the ultra-long C-O bond and charge reorganization to reach a TS (LHd-TS3, Figure 5g) with energy and free energy barriers of 0.26 and 0.19 eV, respectively. In this way, LHc-MS3 dissociates into 2 physisorbed CO_2_ adsorbed on AuBN (LHd-MS4, Figure 5h). During this process, 2 C(O) were oxidized and charges were transferred to Au. As aforementioned, the coadsorbed CO may also act as a spectator in CO_3_ dissociation. For comparison, the dissociation of CO_3_ in LHc-MS2 in this way was also investigated. In this process, the reaction proceeds through a TS (LHc-TS4, Figure 5i) with calculated energy and free energy barriers of 1.85 and 1.59 eV, respectively, to reach the product with a coadsorption of atomic oxygen, CO and a physisorbed CO_2_ around the Au center (LHa-MS1, Figure 3c). The barriers at LHd-TS4 are much higher compared with the direct dissociation of CO_3_ and the reaction of coadsorbed CO with CO_3_, demonstrating the influence of coadsorbed reactants to the evolution of reaction species and the CO conversion.

To this end, the coadsorbed CO, O_2_ or CO_2_ may lead to the formation of new reaction species with different reactivity and their evolution through pathways complementary to the rLH pathway for CO oxidation. Specifically, the coadsorbed CO or O_2_ may promote the evolution of reaction intermediates, considering the CO promoted the dissociation of OCOO (LHa, Figure 3) and O_2_ promoted the dissociation of OCOOCO. Or, they would react to form new reaction species, such as the formation of OCOOCO by the reaction of CO with OCOO (LHb, Figure 4) and the formation of C_2_O_4_ by the reaction of CO with CO_3_ (LHd, Figure 5), initiating new pathways for CO oxidation. Considering the limited E_ad_ and ΔG of these coadsorbed species, their formation would be strongly interfered by reaction conditions. At low partial pressure and high temperature, coadsorption of reactants would be vanished and the reaction may mainly proceed with the rLH pathway (Figure 2) with the formation and dissociation of peroxide intermediate and a direct reaction with gaseous CO. High partial pressure and low temperature may benefit the formation of coadsorbed structures of different reactivity to enable the switching of the reaction pathway. This effect would be significant for the LHb (Figure 3) pathway and promotes the CO-assisted dissociation of OCOO, as well as the CO_3_ formation and evolution initiated with the reaction of AuO with CO_2_ (LHd, Figure 5). CO_3_ is also an important reaction species, considering the strong exothermicity for formation and its reaction with coadsorbed CO, which makes the circumvention of the potentially sluggish reaction of gaseous CO with AuO possible [86].

### 3.4. Comparison with Other Alternative Pathways

We have discussed CO oxidation over AuBN through the rLH pathway and its variants and would now move further to compare these with the remaining. An ER type pathway is initiated with the adsorption and activation of one of the reactants for subsequent reaction with the other gaseous reactants. Theoretically, several ER type pathways were proposed for CO oxidation over SACs, including one molecular ER pathway(ER1), where O_2_ or CO should be activated at the metal center to react with gaseous reactant for the formation of CO_2_ and surface O species [87], two molecular ER(ER2) pathways where gaseous CO react with activated O_2_ forming CO_3_ as a stable intermediate [88] and an ER3 pathway that can be identified with the OCOOCO intermediate formed by the reaction of gaseous O_2_ with two pre-adsorbed CO [53], etc. These pathways all initiate with the van de Waals complexes formed between surface species and the gaseous reactants. According to the thermodynamics data (Figure 1j), AuCO is the most plausible surface species with AuO_2_ + CO ranking the second. The van de Waals complexes formed between preadsorbed CO or O_2_ with gaseous reactants, such as AuO_2_ + CO(g), Au(CO)_2_ + O_2_(g), etc., are less plausible as compared with the corresponding surface species, such as AuO_2_ + CO, etc., while Au(CO)_2_ + O_2_ is unstable and O_2_ may desorb during structure optimization. The superior stability of reaction species on LH type pathways over those van de Waals complexes were previously reported for in CO oxidation over other SACs, where the relative stability among these reaction species were proposed to account for the dominant role of the rLH pathway in the CO oxidation [55,56]. Further to these, the charge transfer from activated O_2_ to CO is required for the formation of CO_3_ intermediate on the ER2 pathway, and a reaction of this kind is always accompanied with high reaction barriers. The ground state of Au(CO)_2_ + O_2_(g) is of triplet symmetry, and the spin is localized on O_2_, making the formation of OCOOCO species of singlet symmetry spin-forbidden. Therefore, the reactions along ER2 and ER3 pathways on AuBN would be rather demanding as compared to those on LH type pathways. As only O_2_ was activated on the ER1 pathway, the reaction is between activated O_2_ and gaseous CO and the calculated energy and free energy barriers for the formation of first CO_2_ are 0.71 and 0.84 eV, respectively, and are much higher as compared with those on the LH type pathways with the involvement of Au.

We fell back to consider the potential formation of the reaction species on ER type pathways and compared them with those on the rLH pathway and newly proposed variants. It is interesting to note that all reaction species, such as CO_3_, OCOOCO, etc., were already included on newly proposed variants of the rLH pathway. Furthermore, the thermostability of some reaction species on ER pathways are lower as compared with corresponding species on LH type pathways, demonstrating that their evolution to those stable species on LH type pathways by adsorbate adsorption/desorption or exchange would be thermodynamics driven. Therefore, their evolution to and on the LH-type pathways would be more reasonable, and would be strongly interfered with by the reaction conditions.

## 4. Conclusions

The reaction condition-dependent catalytic performance of a SAC has long been realized, but seldom investigated before. We investigated CO oxidation pathways over SACs in reaction conditions, using AuBN as a model with extensive first-principles-based calculations. We showed that the adsorption of reactants, namely CO, O_2_ and CO_2_, and their coadsorption with reaction species on AuBN, would be condition dependent, leading to various reaction species with different reactivity and impact to the CO conversion. New pathways originating from these reaction species, complementary to rLH pathway, were proposed and may account for the CO conversion at the corresponding conditions. Specifically, the rLH pathway with CO-mediated activation of O_2_ and the dissociation of the cyclic peroxide intermediate followed by the Eley–Rideal type reduction is dominant at high temperatures, while the coadsorbed CO-mediated dissociation of peroxide intermediate becomes plausible when coadsorption is allowed at low temperatures and high CO partial pressures. Carbonate species would also form in existence of CO_2_ and would react with coadsorbed CO, promoting the CO oxidation. The pathways were currently proposed to investigate CO oxidation on AuBN, but can be delivered to other SACs by further integration with other alternatives pathways that require features not available on AuBN, such as the Mars–van–Krevelen type pathways and those require the involvement of the support. The findings highlight the condition-dependent CO oxidation over SACs may originate from the thermostability of reaction species in detailed conditions and may help to rationalize the current understanding to the superior catalytic performance of SACs.
